# Quantitative humoral profiling of the HIV-1 proteome in elite controllers and patients with very long-term efficient antiretroviral therapy

**DOI:** 10.1038/s41598-017-00759-8

**Published:** 2017-04-06

**Authors:** Wang Zhang, Mohammed M. Morshed, Kajsa Noyan, Aman Russom, Anders Sönnerborg, Ujjwal Neogi

**Affiliations:** 1 Division of Clinical Microbiology, Department of Laboratory Medicine, Karolinska Institutet, Huddinge, Stockholm Sweden; 2 Science for Life Laboratory, Division of Proteomics and Nanobiotechnology, KTH Royal Institute of Technology, Solna, Stockholm Sweden; 3 Department of Medicine Huddinge, Unit of Infectious Diseases, Karolinska Institutet, Karolinska University Hospital, Stockholm, Sweden

## Abstract

A major challenge in evaluating the success of HIV eradication approaches is the need for accurate measurement of persistent HIV during effective antiretroviral therapy (ART). Previous studies have reported that the anti-HIV antibody assay “luciferase immuno-precipitation systems (LIPS)” can distinguish HIV-infected individuals harboring different sizes of the viral reservoirs. We performed antibody profiling of HIV-1 proteomes using LIPS in viremic progressors (n = 38), elite controllers (ECs; n = 19) and patients with fully suppressive long-term antiretroviral therapy (ART) (n = 19) (mean 17 years). IgG was quantified against six HIV-1 fusion proteins: p24, gp41, RT, Tat, integrase and protease. Lower antibody levels to all six-fusion proteins were observed in long-term ART patients compared to viremics (p < 0.05). In contrast ECs had lower antibody levels only against Tat and Integrase (p < 0.05). Principal component analysis and cluster-network analysis identified that 68% (13/19) of the long-term ART patients clustered together with 26% (5/19) ECs. The remaining ECs clustered together with the viremics indicating non-homogeneity among the ECs. The low anti-HIV levels in the long-term treated patients may indicate a restricted remaining viral replication. In contrast, the higher levels in ECs suggest a continuous viral expression with a limited concomitant release of extracellular virus.

## Introduction

A major challenge in the evaluation of human immunodeficiency virus type 1 (HIV-1) eradication approaches is the need for an accurate quantification of any remaining HIV-1. Anti-HIV antibody levels decline when a patient is treated during primary HIV infection (PHI) and may not even develop to fulfill the criteria for HIV infection by standard confirmatory assays^1^. Recently, quantitative humoral profiling of the presumably “cured” Berlin patient revealed no antibodies to several HIV-1 antigens except reverse transcriptase (RT), Tat and gp41^2^. In contrast, the levels persisted to all HIV-1 antigens in most well treated patients. However, a subset of untreated elite controllers (EC) had a similar antibody pattern as the Berlin patient^2, 3^.

Novel accurate high-throughput assays for measurement of the latent reservoir are essential for evaluating eradication strategies. As such, progress towards a cure is certainly hindered by the lack of a robust biomarker for the HIV-1 reservoir. For accurate identification, it is essential to measure conformational epitopes rather linear epitopes. Previous studies have reported that the anti-HIV antibody assay “luciferase immuno-precipitation systems (LIPS)” can distinguish HIV-infected individuals harboring different sizes of the viral reservoirs^2, 4^.

LIPS is a fluid-phase immunoassay that show*s* higher specificity and sensitivity for detection of conformational epitopes than conventional solid-phase ELISA or Western Blot^5–9^. The *Renilla* luciferase fusion protein capable of releasing light is utilized in the LIPS assay making linear detection of antibodies quantitatively for specific antigens. In contrast to solid phase immunoassays, these fusion proteins give an improved precision by utilizing native antigens that target conformational epitopes^7–9^. Therefore, LIPS can be used to screen for humoral response profiling in infectious disease diagnosis^7, 8^, proteome analysis^6^, antibodies in autoimmune disease^9^ and vaccine monitoring.

The aim of present study was to perform antibody profiling of HIV-1 proteomes using LIPS in well-characterized groups of Swedish patients. Although anti-HIV antibody levels decline when a patient is treated during PHI, most patients with four to five years of ART, in whom therapy is initiated during the chronic phase of the infection, have high and stable levels of antibodies^2^. However, we hypothesized that even longer suppressive ART decrease the antibody levels against the HIV-1 proteome because of a further decrease of the expression of viral RNA and proteins. It was also hypothesized that ECs have a restricted viral replication with a low amount of virus-antigen expressed in the reservoirs, leading to lower anti-HIV-antibody levels. We therefore included patients, who had been given 13–20 years of suppressive ART without any detectable viral rebound, and compared the antibody levels with those of ECs and untreated patients with viremia. To the best of our knowledge, our study is the first to provide information about antibody levels to HIV-1 proteomes in very long-term ART experienced patients with fully suppressive therapy who initiated the therapy during chronic phase.

## Results

LIPS detected strong responses against all six HIV-1 antigens (p24, RT, PR, INT, Tat, and gp41) in the samples of the HIV-1 infected patients when compared to the HIV-uninfected controls. The Ruc-antigen fusion HIV-1 constructs are prepared based on HIV-1 subtype B. As Swedish HIV-epidemic is one of the diverse epidemics^10^, we performed cluster analysis and principal component analysis (PCA) using samples from 38 viremic patients representing HIV-1B, HIV-1C, HIV-1A1, CRF01_AE, CRF02_AG, and a novel recombinant BF1. No subtype specific effect was observed with respect to all of the six HIV-1 antigens or the total antibody response (Supplementary Fig. S1).

**Figure 1:**
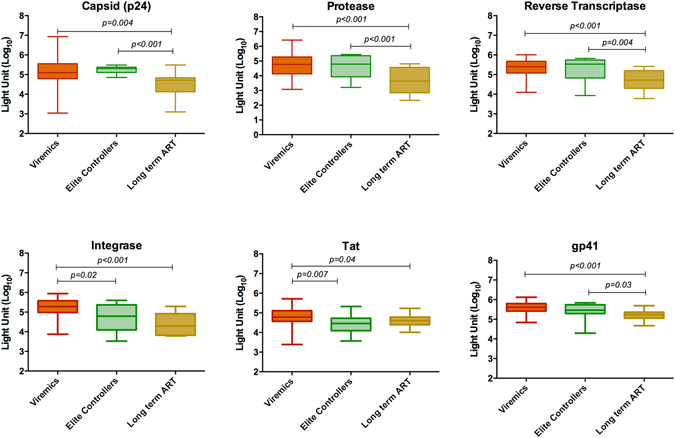
Comparative analysis of the antibody responses among the viremic patients (n = 38), ECs (n = 19) and patients with long-term suppressive ART (n = 19) against capsid (p24), protease, reverse transcriptase, integrase, Tat and gp41. Data indicates a lower response in the long-term suppressive ART group against p24, protease, reverse transcriptase and gp41 compared to the other two groups (Mann Whitney U test; p < 0.05 for all analysis).

**Figure 2:**
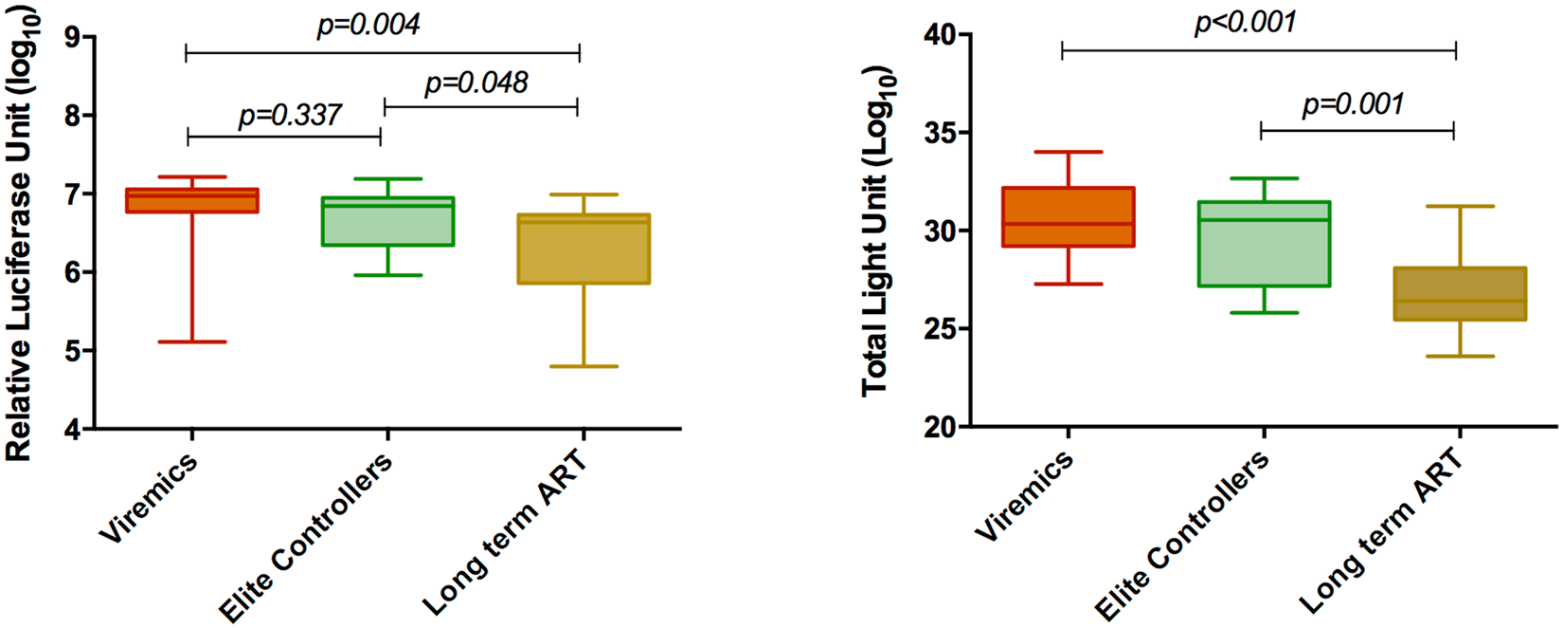
Antibody response against (**A**) 4thG-IA, which quantifies HIV-specific p24 Ag and Ab. Statistically significant lower reactivity was observed in long-term ART patients as compared to the viremics and ECs. (**B**) The total antibody responses measured against all six HIV-1 proteins. Statistically significant lower median total antibody levels were observed in the patients with long-term ART compared to the viremics and ECs.

Lower antibody levels to p24, protease, reverse transcriptase and gp41 were identified in the long-term suppressive ART patients compared to the viremic patients and the ECs (Mann Whitney U test; p < 0.05 for all analysis) (Fig. 1). No statistically significant difference was observed between viremics and ECs. In contrast, statistically significant lower levels of antibodies to Integrase and Tat were found in the ECs and the patients with long-term ART compared to viremics. This data was further supported by the 4th G-IA, which quantifies HIV-specific p24 Ag and Ab (Fig. 2A). We next analyzed the total antibody responses against all six HIV-1 proteins. Statistically significant lower median total antibody levels were observed in the patients with long-term ART compared to the viremics (26.41 vs. 30.34 log_10_ light unit; p < 0.001) and ECs (26.41 vs. 30.41 log_10_ light unit, p = 0.001) (Fig. 2B).

**Figure 3:**
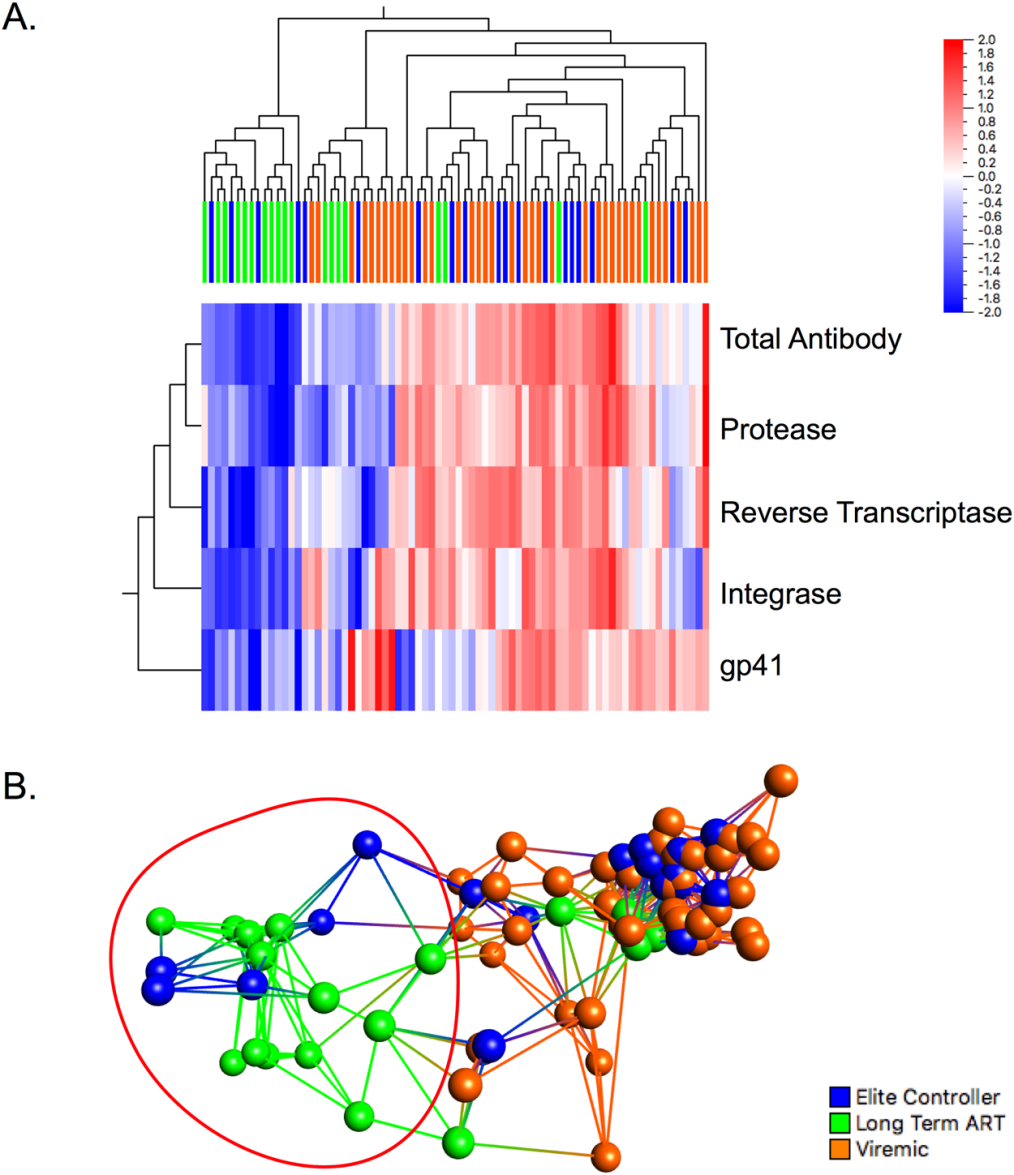
(**A**) The antibody response profiles using hierarchical clustering and (**B**) principal component analysis (PCA) analysis based on the four HIV-1 antigens and the total antibody response. The analysis was performed at a stringent false discover rate (FDR) adjusted p (q) <0.001 using ANOVA.

The antibody response profiles, using principal component analysis (PCA) and hierarchical clustering based on the six HIV-1 antigens and the total antibody response, indicated a greater degree of clustering of patients with long-term ART at false discover rate (FDR) adjusted p (q) less than 0.05 using ANOVA. However, with the more stringent q < 0.001, the clustering of the patients with long-term ART was more prominent based on the humoral responses to the HIV-1 pol antigens (reverse transcriptase, integrase, and protease), gp41 and total antibody (Fig. 3A). The PCA and network analysis identified that 68% (13/19) of the long-term ART patients and 26% (5/19) of the ECs had a direct network or clustered together (Fig. 3B). Most of the ECs (74%; 14/19) clustered however together with the viremic patients.

## Discussion

In this study, we used a modified LIPS assay to quantify the antibody response against six HIV-1 antigens in untreated and treated HIV-1 infected individuals with different disease status. Our data showed that very long-term successful ART is associated with low anti-HIV antibody levels to all tested HIV-1 antigens. In contrast, ECs exhibit lower antibody levels to only Tat and integrase antibody level as compared to viremic patients. The PCA and clustering analysis indicated that the pol antigens are the best predictors of disease and clinical status along with the gp41.

An earlier study has reported that quantitative humoral profiling using LIPS for a sole presumed HIV-1 cure case, “the Berlin Patient”, detected no antibodies against Gag (p24, matrix, nucleocapsid), pol (integrase and protease) and gp120, but low levels were detectable against reverse transcriptase, Tat, and gp41^2^. However, the same study also showed that the antibody levels against the HIV-1 antigens tested were high and stable in most of the patients irrespective of disease or clinical status and no association between low antibody levels and given ART was observed. In contrast, we observed low antibody levels against the entire HIV-1 antigen panel tested in the long-term ART patients’ group. The difference between the two studies is likely to be due to the very long duration of the suppressive therapy in our patients. Thus, unlike the earlier study where the duration of ART was four-five years, our patients were on suppressive therapy for 17–20 years without any detectable viral blips, despite testing for HIV RNA between 2–5 times a year. The LIPS data was further supported by our 4thG immunoassay data. Our data suggest that with prolonged duration of ART a similar decline of antibody response as described in patients who initiated therapy during the acute HIV-1 infection can be obtained^1^. A recent study also suggested that anti-HIV antibody level may correlate to the amount of HIV-1 in the reservoir of HIV-1 treated individuals^4^. We therefore posit that the low levels of the anti-HIV antibody responses in patients with a very long duration of successful ART correspond to limited antigen expression due to a decreased of latent HIV-1 reservoir. Based on our PCA and network analysis, we further support that the antibody response against enzymes involved in HIV-1 replication i.e. pol proteins (reverse transcriptase, protease and integrase) might be better surrogate markers than the structural proteins (e.g Gag or Env proteins) of the disease state as well as the size of the reservoir as suggested recently^4^.

We hypothesized at the beginning of the study that ECs would express low levels of anti-HIV antibody levels due to a limited HIV-1 reservoir. However, the heterogeneity in antibody levels among ECs suggested that this category of patients is not a homogenous group, which is in line with previous study^2^, despite that our ECs all fulfilled stringent criteria. A subset of ECs had low levels of antibodies against p17, and reverse transcriptase, integrase, and/or protease compared to the non-controllers. Statistically significant lower antibody levels in ECs were however only observed with integrase and tat proteins compared to the viremic patients.

While comparing the viremics and the ECs, only integrase and Tat antibody levels were significantly lower in ECs. The data that our ECs had low anti-integrase antibody levels could possibly be related to a lower number of integration events and a lower activity of the integrase enzyme associated with a very low level viremia. The extracellular release of Tat is apoptosis independent^11^ and activates virus replication and cellular gene expression, increasing virus transmission to neighbor cells^12^. Lower release of extracellular Tat, and thereby low anti-Tat antibody levels, could therefore be associated with lower replication and a decreased cell-to-cell transmission of viruses in the ECs as well as in long-term ART patients^13^.

The present study has some limitations and strengths that merit comments. First, this is a cross-sectional study that limits analysis of the dynamics of the antibody responses in the ECs and treated patients. Second, the number of ECs and long-term ART patients are relatively low. However ideal ECs are rare and we used strict criteria to select the patients with long-term successful ART. The major strength is the detailed knowledge of the cohort and the selected patients resulting in a high quality of the data. We used thus nearly 20 years of patients’ clinical and demographic data to select these patients.

In conclusion, our findings show that anti-HIV antibody responses are low to the whole HIV-1 proteome after a very long duration of successful ART, which is in line with a limited antigen expression and a limited latent HIV-1 reservoir. The heterogeneity in antibody levels among ECs suggests that this category of patients is not a homogenous group despite our strict criteria. Therefore, ECs may not be the best model of a functional cure^14^ and a more strict definition of ECs should be applied in order to describe such patients. Instead, long-term ART patients without rebound in viremia could instead potentially be the best interventional models. Further studies could aim at performing longitudinal studies to identify the dynamics of the antibody responses among the ECs and long-term ART patients.

## Methods

### Patients

Plasma samples were obtained from three categories of HIV-1 infected patients; therapy naïve patients with viremia (n = 38), patients with long-term ART without detectable viremia (n = 19), and untreated elite controllers (ECs; n = 19) with undetectable virus and stable CD4 cells; as well as from HIV-1 negative individuals (n = 17) (Table 1). The ECs were defined according the EuroCoord definition^15^ i.e. either known HIV-1 positivity for ≥1 year and ≥3 consecutive viral load (VL) <75 copies/ml over a year (and all previous VLs <1000 copies/ml or known HIV-1 positivity ≥10 year and minimum two VL measurements of which ≥90% of all VLs <400 copies/ml. At the time of sampling the mean (SD) viral load for HIV-1 seropositive viremics was 4.68 (0.75) log_10_ copies/mL with median (IQR) CD4^+^ T-cell count of 267 (137–520) cells/mm^3^. The median (IQR) CD4^+^ T-cell count for the ECs was 950 (695–1655) cells/mm^3^ and for the patients with long-term suppressive ART 550 (490–610) cells/mm^3^. The mean duration since HIV diagnosis for the ECs were 12 years (range: 3–33 years) and for long-term ART group 22 years (range: 13–31 years). The mean duration of treatment was 17 years (range: 13–20). Detailed patient characteristics of the patients are given in Supplementary Tables S1 and S2, respectively.

**Table 1:** Patients’ demographic and clinical characteristics.

Parameters	Healthy controls		HIV-1 seropositive patients*		
		Viremics	Elite Controllers	Long Term ART	P values
N	17	38	19	19	
Age; Median (IQR)	49 (27–65)	34 (31–45)	35 (25–42)	31 (25–32)	0.06
Gender; Female; N (%)	8 (47)	22 (57)	9 (47)	3 (16)	0.005
Route of Transmission; N (%)					
Heterosexual	NA	25 (66%)	10 (52%)	10 (52%)	0.75
MSM		8 (21%)	4 (21%)	5 (26%)	
PWID		3 (8%)	2 (11%)	3 (16%)	
Other/Unknown		2 (5%)	3 (16%)	1 (5%)	
Country of Birth; N (%)					
Sweden	NA	17 (45%)	8 (42%)	13 (68%)	0.18
Abroad		21 (55%)	11 (58%)	6 (32%)	
Country of transmission; N (%)					
Sweden	NA	21 (55%)	8 (42%)	6 (32%)	0.22
Abroad		17 (45%)	11 (58%)	13 (68%)	
Subtypes; N (%)**					
A1	NA	6 (16%)	1 (5%)	0	NA
B		9 (23%)	2 (10%)	2 (10%)	
C		16 (42%)	7 (37%)	1 (5%)	
Other		7 (18%)	2 (10%)	1 (5%)	
ND		0	7 (37%)	15 (79%)	
*Parameter at time of sampling*					
Duration since HIV diagnosis Mean (Range), years	NA	0.09 (0–1.5)	12 (3–33)	22 (13–31)	<0.001
Duration of treatment Mean (Range), years	NA	NA	NA	17 (13–20)	NA
CD4+ T-cells; cells/mm^3^ Median (IQR)	NA	267 (137–520)	950 (695–1655)	550 (490–610)	<0.0001
CD4+ T-cells %, Median (IQR)	NA	20 (13–27)	46 (33–48)	34 (29–39)	<0.001
CD8+ T-cells; cells/mm^3^ Median (IQR)	NA	760 (530–980)	780 (590–905)	580 (450–780)	0.26
CD8+ %, Median (IQR)	NA	51 (42–63)	33 (27–45)	39 (31–46)	<0.001
CD4: CD8	NA	0.42 (0.2–0.74)	1.48(0.79–1.71)	0.86 (0.70–1.13)	<0.001
Plasma HIV-1 RNA, log_10_ copies/mL, Mean (SD)	NA	4.68 (0.75)	<20	<20	NA

Viremics = untreated patients with detectable viremia; Long Term ART: patients who have received antiretroviral therapy without any viral blips or virological failure since initiation of therapy; NA: Not applicable. **Some of ECs subtyping was based on the Gag or LTR region.

### Preparation of Ruc-HIV-antigen for Luciferase immunoprecipitation systems (LIPS)

LIPS assay was performed in a modified version compared to what was originally reported^16^. Ruc-antigen fusion HIV-1 constructs for p24, reverse transcriptase (RT), integrase (INT), protease (PR), trans-activator of transcription (Tat) and gp41 were used (kind gift from Peter D Burbelo, NIH, US). HEK293 cells were transfected with each of the constructs using FuGENE® HD Transfection Reagent (Promega, WI, US) and harvested after 48 hours in lysis buffer available in *Renilla* Luciferase Assay System kit (Promega, WI, US). The cells were then sonicated in Branson Sonifier 150 followed by centrifugation at 15,000xg for 4 minute at 4 °C. The crude lysate was then used to measure the light unit (LU). After diluting 1 μl of the lysate with 9 μl of phosphate buffer saline (PBS) in a well of 96-well plate, 50 μl of Renilla Luciferase Assay Reagent was added and immediately the luminescence was measured using luminometer (Infinite® 200 PRO, Tecan).

### LIPS assay

The protein A/G IgG binding buffer (50 μl) (ThermoFisher, US) was added to each well of the 96-well plate followed by addition of 1 μl of patients’ plasma of patient. After mixing, 1 × 10^7^ light units (LU) of Ruc-HIV-antigen extract were added in each well and the plate was incubated for one hour at room temperature on a rotor shaker. After the incubation, 5 μl of a 30% suspension of protein A/G magnetic beads (ThermoFisher, US) were added in each well and incubated for one hour at room temperature. After incubation, a Magnetic Stand (ThermoFisher, US) was placed below the plate and waited for two minutes to magnetize all the beads on the bottom of each well. The supernatant was discarded and the plate was washed three times using 100 μl protein A/G IgG binding buffer (ThermoFisher, US). Infinite® 200 PRO series microplate luminometer was used for determining luminescence in each well using a single injector. Renilla Luciferase Assay Reagent (Coelenterazine substrate) was prepared as described by the manufacturer. After priming, 50 μl of coelenterazine substrate was injected and the plate was shaken for 5 sec, followed by a 5 sec read of luminescence. The assay was performed in duplicates in two biological replicates. Each plate has a positive control (known LU value of each protein tested) and a negative control (beads and extract). The LU data presented are corrected for background by subtracting the LU of the negative control and log_10_ transformed.

### Fourth-generation (4thG) antigen (Ag)/antibody (Ab) immunoassay (IA)

ARCHITECT HIV Ag/Ab Combo (Abbot) was used to quantify HIV-specific p24 Ag and Ab. The sample relative luciferase unit (RLU) was calculated using multiplying the sample to cut off ratio with cut off RLU. The cut-off RLU was obtained multiplying calibrator 1 mean RLU with 0.40.

### Statistical Analysis

GraphPad Prism (San Diego, CA) was used for statistical analyses. Antibody levels were reported as median levels with interquartile range (IQR). The nonparametric Mann–Whitney *U* test was used for comparison of the different groups. Principal component analysis (PCA) and differential profile (heatmap) of the antibody responses against the six HIV-1 fusion proteins were analyzed using Qlucore Omics Explorer version 3.2. The PCA was performed at false discover rate (FDR) adjusted p (q) <0.05 and stringent <0.001 using ANOVA.

### Ethical considerations

The study was approved by regional ethics committees of Stockholm (2013/1944–31/4) and all the methods were performed in accordance with approved institutional guidelines. All participants gave informed consent. The patient identity was anonymised and delinked prior to analysis.

## Electronic supplementary material

Table S1, S2; Figure S1
