# Supplementary material for: Quantitative humoral profiling of the HIV-1 proteome in elite controllers and patients with very long-term efficient antiretroviral therapy
Source: Sci Rep. 2017 Apr 6;7:666. doi: 10.1038/s41598-017-00759-8 (PMC5429677; doi:10.1038/s41598-017-00759-8)

Quantitative humoral profiling of the HIV-1 proteome in elite controllers and patients' with very long-term efficient antiretroviral therapy

Wang Zhang[1,2], Mohammed M. Morshed[1,] Kajsa Noyan[1], Aman Russom[2], Anders Sönnerborg[1,3], Ujjwal Neogi[1,*]

[1]Division of Clinical Microbiology, Department of Laboratory Medicine, Karolinska Institutet, Huddinge, Stockholm, Sweden

[2]Science for Life Laboratory, Division of Proteomics and Nanobiotechnology, KTH Royal Institute of Technology, Solna, Stockholm, Sweden.
[3]Department of Medicine Huddinge, Unit of Infectious Diseases, Karolinska Institutet, Karolinska University Hospital, Stockholm, Sweden

Table S1. Characteristics of 19 elite controllers (ECs) in whom the plasma HIV RNA load has been undetectable in the absence of antiretroviral treatment.

Patient	Age	Gender	Ethnicity	Inclusion criteria*	Mode of Transmission	No. of years of HIV infection	CD4 count, median (min-max)	CD4%, median (min-max)	CD8 count, median (min-max)	CD8%, median (min-max)	CD4/CD8 ratio, median (min-max)
**#1**	62	Male	Caucasian	2	Heterosexual	9	550 (380-610)	28 (21-30)	950 (660-1490)	48.5 (45-60)	0.59 (0.37-0.62)
**#2**	52	Male	Black	1	Heterosexual	3	955 (630-1180)	41 (33-46)	745 (100-860)	35 (34-39	1.18 (0.84-1.37)
**#3**	46	Female	Black	1	Heterosexual	14	653 (520-820)	42 (37.5-47)	490 (390-545)	32.5 (30.5-35)	1.32 (1.18-1.49)
**#4**	41	Female	Black	1	Heterosexual	5	765 (630-980)	31 (28.5-33)	1068 (900-1180)	42.3 (40.5-47)	0.72 (0.65-0.83)
**#5**	40	Female	Black	1	Heterosexual	3	755 (660-790)	47 (45-47.5)	528 (480-610)	33.5 (32.5-35)	1.41 (1.3-1.46)
**#6**	44	Female	Black	1	Heterosexual	19	645 (508-880)	32 (27-38)	855 (730-1010)	47 (41-52)	0.65 (0.59-0.92)
**#7**	51	Male	Caucasian	1	MSM	11	1258 (950-1354)	47 (42-50)	843 (610-986)	32 (30.5-35)	1.52 (1.22-1.59)
**#8**	56	Male	Caucasian	1	PWID	29	730 (400-1130)	39 (23-48)	559 (305-1025)	28.5 (21-40)	1.37 (0.68-1.9)
**#9**	68	Male	Caucasian	1	MSM	6	1040 (801-1210)	46 (43-48)	657 (570-680)	28 (26-35)	1.68 (1.23-1.83)
**#10**	45	Male	Caucasian	1	Blood product	33	714 (590-962)	36.5 (27.5-44)	649 (433-1352)	30 (21.7-56.5)	1.21 (0.49-1.73)
**#11**	49	Male	Black	2	Heterosexual	11	673 (520-810)	21 (19-22)	2103 (1410-2450)	64 (60-65)	0.33 (0.3-0.36)
**#12**	55	Male	Caucasian	2	MSM	29	930 (778-1268)	43 (34-47)	621 (498-888)	27.3 (24-34)	1.49 (1.02-1.96)
**#13**	32	Female	Black	1	Blood product	8	959 (777-1240)	43.3 (38-47)	610 (610-610)	30 (30-30)	1.32 (1.32-1.32)
**#14**	48	Male	Latin	1	MSM	4	995 (870-1270)	39.5 (37-45.5)	885 (790-965)	34.3 (34-35)	1.1 (1.1-1.3)
**#15**	28	Female	Black	1	Unknown	5	947 (792-1096)	37 (33-40.5)	949 (810-1072)	34 (32.5-37.5)	1.07 (0.95-1.26)
**#16**	40	Female	Black	1	Heterosexual	5	606 (504-695)	45.8 (43-48)	403 (367-439)	35.4 (35.3-35.5)	1.34 (1.1-1.55)
**#17**	43	Female	Caucasian	1	Heterosexual	14	1096 (636-1535)	45 (38-57.5)	827 (530-1058)	33 (28-38)	1.42 (1.05-2.31)
**#18**	49	Female	Caucasian	1	PWID	6	1334 (1095-1620)	45.8 (39.5-48-5)	1000 (765-1358)	32.5 (28-44)	1.44 (0.9-1.68)
**#19**	36	Male	Black	2	Heterosexual	12	1320 (921-1810)	60 (54-62)	511 (430-610)	23 (20-26.5)	2.69 (1.05-3.22)

NOTE. MSM, men who have sex with men; PWID, people who inject drugs; ND, not determined; * HIV RNA levels <75 copies/ml on minimum 3 consecutive determinations, spanning over at least 12-months ART-free period with all previous determinations below 1000 copies/ml (**1**) or HIV positive status for ≥10 years with minimum 2 HIV RNA level determinations, ≥90% of all HIV RNA determinations below 400 copies/ml (**2**).

Supplementary Table S2: Characteristics of 19 patients with long-term antiretroviral treatment.

**Patient**	**Age**	**Gender**	**Ethnicity**	**Mode of Transmission**	**No. of years of HIV infection**	**Years on ART**
**#1**	49	Male	Black	Unknown	17	17
**#2**	52	Male	Black	Heterosexual	20	20
**#3**	49	Female	Caucasian	Heterosexual	25	19
**#4**	49	Male	Black	Heterosexual	23	16
**#5**	62	Male	Caucasian	IVDU	30	*20*
**#6**	68	Male	Caucasian	Heterosexual	20	16
**#7**	56	Male	Caucasian	MSM	30	*16*
**#8**	55	Male	Caucasian	IVDU	26	*15*
**#9**	62	Male	Caucasian	Heterosexual	22	20
**#10**	51	Male	Caucasian	Heterosexual	18	*18*
**#11**	62	Male	Caucasian	MSM	13	*13*
**#12**	51	Male	Caucasian	Heterosexual	19	19
**#13**	41	Female	Black	Heterosexual	16	*16*
**#14**	45	Male	Caucasian	Heterosexual	17	*16*
**#15**	48	Male	Caucasian	MSM	17	*17*
**#16**	56	Male	Caucasian	MSM	31	*20*
**#17**	53	Male	Caucasian	MSM	28	15
**#18**	40	Female	Asian	Heterosexual	19	19
**#19**	68	Male	Caucasian	IVDU	27	*16*

Figure S1. Effect of HIV-1 subtype in determining the Ab response using modified LIPS-assay. Cluster analysis and principal component analysis (PCA) using samples from 38 viremic patients representing HIV-1B, HIV-1C, HIV-1A1, CRF01_AE, CRF02_AG and a novel recombinant BF1.

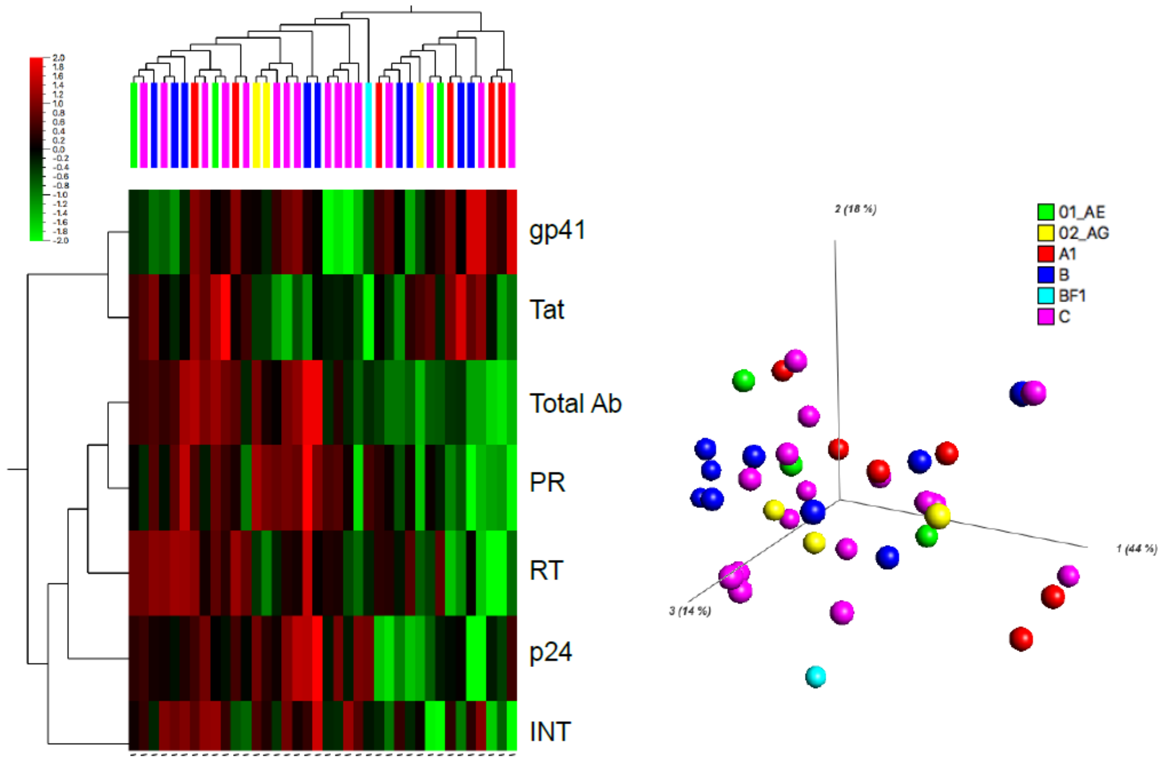

Supplement: Supplementary file 1 — Table S1, S2; Figure S1 [file 41598_2017_759_MOESM1_ESM.pdf]
